# The predictive value of serum lipids for eye metastases in male nasopharyngeal carcinoma patients

**DOI:** 10.1042/BSR20201082

**Published:** 2020-06-25

**Authors:** Zhen Xie, Yi Shao

**Affiliations:** Department of Ophthalmology, The First Affiliated Hospital of Nanchang University, Jiangxi Province Ocular Disease Clinical Research Center, Nanchang 330006, Jiangxi, People’s Republic of China

**Keywords:** eye metastasis (EM), male, nasopharyngeal carcinoma (NPC), risk factor, total cholesterol (TC), triglyceride (TG)

## Abstract

**Background**: Nasopharyngeal carcinoma (NPC) is a tumor that is commonly found in southern China. NPC has several risk factors, such as infection with the Epstein–Barr virus. However, we know little about the risk factors for eye metastasis (EM) in male patients with NPC. Serum lipids are well recognized as risk factors for cardiovascular disease, and recent studies show that they also have a relationship with the development of NPC.

**Purpose**: We designed the present study to determine whether they were relevant with the development of EM in male NPC patients by detecting the levels of several serum lipids.

**Methods:** A total of 1140 male patients with NPC were enrolled in this retrospective study and we divided them into two groups: the metastasis (EM) group and non-eye metastasis (NEM) group. A variety of serum lipids between the two groups were tested and compared.

**Results**: There were statistical differences in the levels of serum TG and TC between these two groups. Binary logistic regression showed that TG and TC were independent risk factors for EM in male NPC patients with *P*=0.004 and *P*<0.001, respectively. The area under the curve of TG and TC were 0.764 and 0.681, respectively, using cutoff values of 0.975 and 3.425 mmol/l, respectively. We found that TG had higher sensitivity and specificity values with 87.5% and 62.7%, respectively, than TC which were 50.0% and 87.2%.

**Conclusion:** TG and TC are potential risk factors for eye metastases in male NPC patients.

## Introduction

Nasopharyngeal carcinoma (NPC) is one of the most prevalent malignant tumors in the world and has high mortality in some parts of the world, especially in Southeast Asia, including China. It has been reported that Guangdong province in southern China had about 20–40 cases per 100,000 inhabitants, which is the most popular area of NPC in the world [1]. Epstein–Barr virus (EBV) infection, environmental factors (especially diet), gender, and genetic susceptibility contribute to the development of NPC [2]. NPC is associated with numerous symptoms such as stuffy nose, bloody nose, stuffy ears, sense of hearing loss, headache, and some with ocular symptoms such as diplopia, which makes diagnosis challenging [3]. Distant metastatic sites are usually multifocal, and the lungs, bones, and distant lymph nodes are the most common among these [4–6]. Once metastasis occurs, prognosis is usually poor; for example, the survival time from diagnosis for skin metastasis is shorter than nine months [7]. A pediatric nasopharyngeal carcinoma with retinal metastasis had been reported first time in 2009 [8] and a previous 18-year period study founded that NPC was the most common primary cancer that metastasizes to the orbit in southern China [9]. Although the incidence of eye metastasis (EM) is lower than that of lung and bone metastasis, it is becoming increasingly common and is indicative of poor prognosis.

Radiotherapy is considered to be the standard treatment for NPC since NPC is sensitive to radiotherapy. Currently, radio-chemotherapy (RCT) has been extensively applied to advanced NPC patients with distant metastasis to improve healing [10]. However, for patients with distant metastasis, the survival rate is low because NPC is difficult to diagnosis at an early state due the lack of specificity. Plasma EBV DNA is one of the most well-recognized biomarkers for detecting NPC and monitoring the progression of the disease [11,12]. At present, CT, MRI, and other radiological examinations are also a major diagnostic method, which are expensive, time-consuming and even invasive. In addition, their limitations in sensitivity and specificity suggest a convenient diagnostic technique with high accuracy is urgently needed, for example, discovery of molecular markers. Detecting metastasis as the main cause of death with high specificity and sensitivity is of vital importance.

Blood lipid levels have been known to have epidemiologic relationships with cardiovascular disease. However, recently, it has been shown that lipid levels have a close relationship with cancer development [13–15]. Importantly, some specific serum lipids have been used already to help the clinical diagnose and treatment in tumors [16–18]. Therefore, we collected data from male patients with NPC eye metastasis and evaluated the levels of some serum lipids in order to further explore their relationship with eye metastases in male NPC patients.

## Materials and methods

### Study design

We enrolled 1140 male patients with NPC in this retrospective study and divided them into two groups: the metastasis (EM) group and non-eye metastasis (NEM) group. Computed Tomography (CT) and magnetic resonance imaging (MRI) were used to clarify a diagnosis. Following were the inclusion criteria for EM group: (1) excluded primary eye malignant tumors, for example, rhabdomyosarcoma and neuroblastoma; and (2) excluded benign eye tumors, for example, dermoid cysts, vascular, and neurogenic benign tumors an orbital inflammatory pseudotumor. NEM patients were excluded if they had an eye lesion according to eye examinations.

### Data collection

Basic demographic information and relevant clinical data were collected according to their medical records, including age, height, weight, address, histological types, and metastasis sites. What’s more, the levels of detected serum lipids, including triglyceride (TG), total cholesterol (TC), high-density lipoprotein (HDL), low-density lipoprotein (LDL), apolipoprotein A1 (Apo A1), and apolipoprotein B (Apo B).

### Statistical analyses

We used Student’s *t*-tests, and Chi-squared tests to evaluate whether there were statistical differences in age and histopathology between the EM and EOM groups. The levels of detected serum lipids, namely, the independent risk factors were investigated by building binary logistic regression models. Receiver operating characteristic (ROC) curves were created and the area under the curve (AUC) was calculated to assess their diagnostic value and accuracy as EM predictions. We chose *P*-value <0.05 as statistical significance. Statistical analyses were performed accurately by Microsoft Office Excel 2018 software (Microsoft Corporation, U.S.A.) and SPSS version 19.0 software (SPSS Inc., Chicago, IL, U.S.A.).

## Results

### Demographics and clinical characteristics

**Figure 1:**
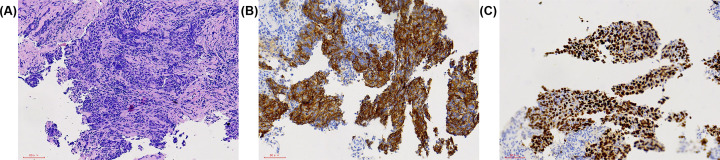
The HE staining and IHC images from nasopharyngeal carcinoma patients with eye metastasis **Notes:** (**A**) Nasopharyngeal carcinoma (HE×200), (**B**) P40(+) (SP×200), (**C**) CK (+) (SP×200). The tissue was collected from eye metastasis site of nasopharyngeal carcinoma.

**Figure 2:**
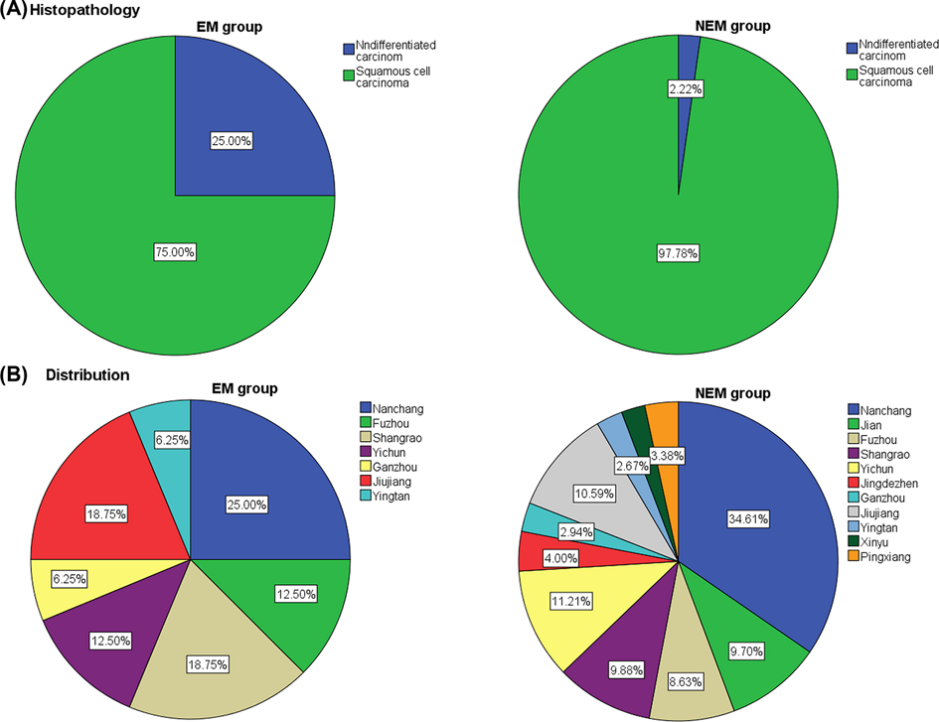
Histopathology (A) and Distribution (B) of male nasopharyngeal carcinoma patients with or without eye metastasis **Abbreviations:** EM, eye metastasis; NEM, non-eye metastasis.

We enrolled totally 1140 male patients in the present study, including 16 cases of EM and 1124 cases of NEM. Student’s *t*-tests and Chi-squared test did not indicate statistical differences between the EM and NEM groups in terms of age and histopathology. The mean age of the EM and NEM groups was similar at 57.8 ± 9.3 and 54.0 ± 8.2 years, respectively and squamous cell carcinoma was the most prevalent histopathological type (1111 cases, 97.5%). Table 1 contains more detailed demographics and clinical characteristics of all male patients with NPC in the present study. The HE staining and IHC images from nasopharyngeal carcinoma patients with eye metastasis are shown in Figure 1. We can conclude from Figure 2 that majority of them came from Nanchang, the capital of Jiangxi Province. We also observed that the most common metastasis in the NEM group was to the cervical lymph nodes. Patients with advanced cancer usually had a greater risk of metastasis, according to our data, most patients with eye metastasis had lymph node metastasis and skull base bone infiltration invasion, which was an indicator of advanced cancer.

**Table 1:** The clinical characteristics in EM and NEM groups

Characteristics	EM (%) (*n*=16)	NEM (%) (*n*=1124)	*P* value
Mean age^*^	57.8 ± 9.3	54.0 ± 8.2	0.072
Histopathology^†^			
Squamous cell carcinoma	12 (75.0)	1099 (97.8)	0.122
Undifferentiated carcinoma	4 (25.0)	25 (2.2)	
Distribution			
Nanchang	4 (25.0)	389 (34.6)	
Jian	0	109 (9.7)	
Fuzhou	2 (12.5)	97 (8.6)	
Shangrao	3 (18.8)	111 (9.9)	
Yichun	2 (12.5)	126 (11.2)	
Jingdezhen	0	45 (4.0)	
Ganzhou	1 (6.3)	33 (2.9)	
Jiujiang	3 (18.8)	119 (10.6)	
Yingtan	1 (6.3)	30 (2.7)	
Xinyu	0	27 (2.4)	
Pingxiang	0	38 (3.4)	

*Student’s *t*-test was performed.

†Chi-square test was used. *P*<0.05 revealed significant difference.

**Abbreviations:** EM, eye metastasis; NEM, non-eye metastasis.

### Clinical data and risk factors of EM

The levels of the following serum lipids were determined for the NPC patients: triglyceride (TG), total cholesterol (TC), high-density lipoprotein (HDL), low-density lipoprotein (LDL), apolipoprotein A1 (Apo A1), and apolipoprotein B (Apo B). The results indicated that the mean levels of TG in EM group and NEM group were 0.8 ± 0.3 and 1.4 ± 1.1 mmol/l, respectively. Moreover, the levels of TC in EM group and NEM group were 3.3 ± 1.5 and 4.4 ± 1.2 mmol/l, respectively. Importantly, both differences had statistical significance (*P*<0.05). No statistical difference was shown in the concentrations of HDL, LDL, Apo A1, and Apo B (*P*>0.05; Table 2). The binary logistic regression results illustrated that the levels of TG and TC were potential independent risk factors for EM (Table 3).

**Table 2:** Comparison of tumor makers between EM and NEM group

Tumor marker	EM	NEM	*P* value
TG (mmol/l)	0.8 ± 0.3	1.4 ±1.1	<0.001
TC (mmol/l)	3.3 ± 1.5	4.4 ± 1.2	0.017
HDL (mmol/l)	1.3 ± 0.5	1.3 ± 0.5	0.894
LDL (mmol/l)	14.8 ± 48.9	2.8± 2.5	0.343
Apo A1(g/l)	1.7± 0.4	1.8 ± 1.4	0.918
Apo B (g/l)	1.4 ± 1.5	1.1± 0.6	0.468

**Note:** Student’s *t*-test test was performed. P<0.05 revealed statistical significance.

**Abbreviations:** Apo A1, apolipoprotein A1; Apo B, apolipoprotein B; EM, eye metastasis; HDL, high-density lipoprotein; LDL, low-density lipoprotein; NEM, non-eye metastasis; TC, total cholesterol; TG, triglyceride.

**Table 3:** The binary logistic regression model between EM and NEM group

Tumor marker	*B*	Exp(*B*)	*P* value
TG	2.486	12.010	0.004
TC	0.826	2.285	<0.001

**Note:** The binary logistic analysis was performed. *P*<0.05 means significant difference.

**Abbreviations:** B, coefficient of regression; TC, serum total cholesterol; TG, triglyceride.

### Cut-off value, sensitivity, specificity, and AUC of TG and TC for the diagnosis of EM

**Figure 3:**
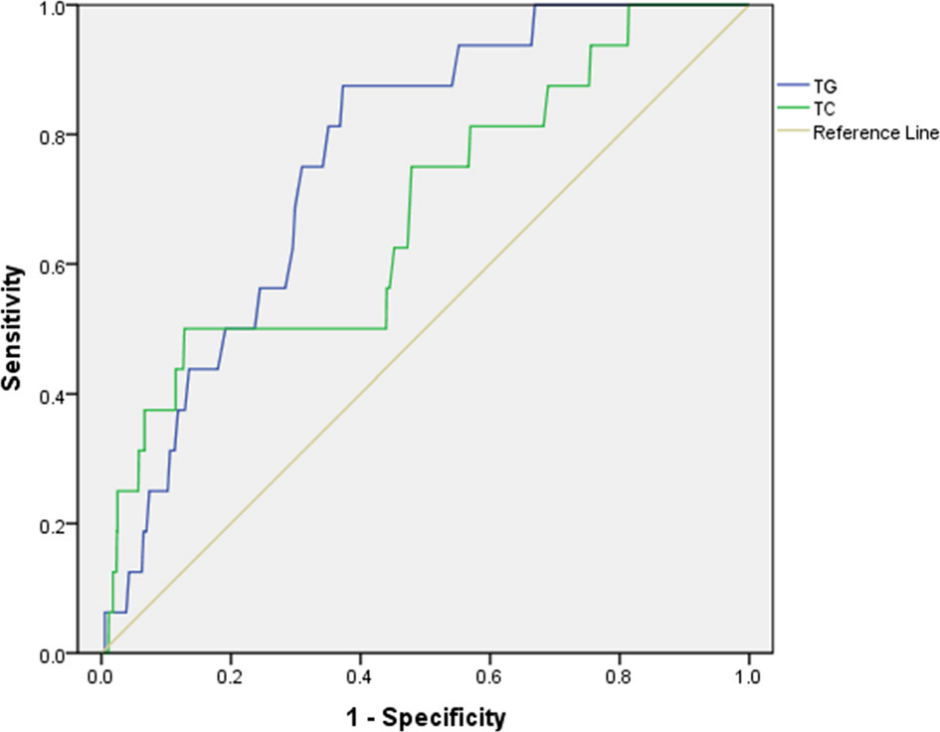
The ROC curves of different markers to predict EM in primary lung cancer The ROC curves showed that the area under the ROC curve of TG and TC were 0.764 and 0.681 (*P*=0.004, *P*<0.001, respectively). **Abbreviations:** ROC, receiver operating characteristic; TC total cholesterol; TG, triglyceride.

In the present study, the ROC curves for TG and TC were mapped to determine their diagnostic value and accuracy for male patients with NPC (Figure 3). The ROC curves indicated that the AUC of TG and TC were 0.764 and 0.681, respectively, using cutoff values of 0.975 and 3.425 mmol/L, respectively. We found that TG had higher sensitivity and specificity values with 87.5% and 62.7%, respectively, than TC which were 50.0% and 87.2%. Table 4 shows more details about the cutoff value, sensitivity, specificity, and AUC values for TG and TC.

**Table 4:** The cutoff value, sensitivity, specificity and AUC of TG and CYFRA21-1 in detecting the EM in metastatic NPC

Tumor maker	Cutoff value	Sensitivity	Specificity	AUC	*P* value
TG (mmol/l)	0.975	87.5%	62.7%	0.764	<0.001
TC (mmol/l)	3.425	50.0%	87.2%	0.681	0.013

**Note:** The sensitivity and specificity were calculated at the point of Youden's index. P<0.05 indicates statistical significance.

**Abbreviations:** AUC, area under the curve; EM, eye metastasis; TC, total cholesterol; TG, triglyceride; NPC, nasopharyngeal carcinoma.

## Discussion

Because the nasopharyngeal cavity is deep and concealed when NPC happens, it is difficult to observe until it spreads to adjacent structures or lymphatic metastasis occurs. In addition, without specific symptoms, nosebleeds or a stuffy nose, sputum with blood, earplugs, tinnitus, hearing disorders, swelling of the neck due to enlarged lymph nodes, eyelid drooping, diplopia, facial paralysis as well as headaches can appear as symptoms [19]. Studies have shown that Chinese people from Hong Kong or southern China are the highest risk group for NPC. People from North Africa and the Middle East also have moderate risk for NPC. In Singapore, men are three times more likely to develop NPC than women. Therefore, NPC is perceived as having a particularly high fatality rate for men in southern China [20–23]. We designed the present study to include Chinese male NPC patients with the aim of finding potential risk factors that can specifically monitor NPC, which is of clinical significance.

NPC has a lower risk of EM than bone and lung metastases, but the rate for EM is increasing. EMs occur with different cancers and are usually considered as a sign of a poor prognosis [24–26]. In the late stage, NPC invades the optic nerve that is close to the normal optic chiasm, causing vision loss, nasal or temporal hemianopia, and blindness in one or both eyes. It is common for patients to see a doctor because of orbital pain, extraocular paralysis, and exophthalmos [3,27]. However, since the size of a metastatic lesion is usually small, it is difficult to distinguish the ocular symptoms of NPC patients by conventional imaging detection. Early diagnosis of EM in NPC patients is not easy, but it is of vital significance. At present, radiotherapy is a major treatment, but this will inevitably cause eye injury such as a radiation cataract and damage to the retina and optic nerve, which makes it hard to distinguish from the original EM [28–31]. There is no doubt that finding an effective detection index of NPC eye metastasis to provide different etiology treatments will greatly improve the prognosis of patients.

With the development of research, serum lipids as serological markers have been widely used in clinical practice. Compared with traditional diagnostic techniques, such as MRI and CT, which are time-consuming, expensive, and limited in sensitivity, detecting serum tumor markers has a great advantage. Previous studies on serum lipids as risk factors for different cancers are shown in Table 5 [16,32–37].

**Table 5:** The serum lipids as risk factors of different kinds of cancer

Author	Year	Cancer	Serum lipids
Sun et al. [32]	2019	Gastric cancer	TG, HDL
Dashti et al. [33]	2019	Colorectal cancer	TC, TG
Zhang et al. [16]	2017	Non-small-cell lung cancer	TC, DB
Arthur et al. [34]	2016	Prostate cancer	TG, TC
Kapil et al. [35]	2013	Breast cancer	TG, TC
Saito et al. [36]	2013	Liver cancer	LDL
Lyu et al. [37]	2018	Lung cancer in men	TC

**Abbreviations:** DB, direct bilirubin; HDL, high-density lipoprotein; LDL, low-density lipoprotein; TC, total cholesterol; TG, triacylglycerol.

To identify the risk factors for EM in NPC, we collected data on serum lipids levels of male NPC patients. By detecting the levels of serum TC, TG, HDL, LDL, Apo A1, and Apo B, we illustrated that TC and TG can be taken as potential risk factors for EM in male NPC patients with lower levels for both in the EM group.

The main function of triglyceride (TG) is to supply and store energy and to fix and protect the viscera. There are two main sources of TG. They are exogenously obtained from fat in food and endogenously synthesized by the liver and adipose tissue. There are gender and age differences in the level of TG. Usually TG levels rise with age, and women have more stable TG levels than men [38]. Ulmer et al. conducted a health survey for 10.6 years and collected serum TG levels in 156,153 patients with malignant tumors. Higher TG levels were found related with increased risk of rectal, lung, and thyroid cancer in men and women. Nevertheless, there was a negative correlation between serum TG levels and non-Hodgkin’s lymphoma. Male TG levels had a negative correlation with prostate cancer and had a positive correlation with kidney cancer. In women, TG levels were positively correlated with gynecological cancer [14]. In our study, TG levels of male NPC patients with EM are reduced compared with NEM patients, and the mechanism is unclear. Thus, the differences in TG levels with different cancer types require further study.

As an essential material for tissues and cells, cholesterol plays a vital role in the function of cell membranes. Moreover, it can transform into bile acids, vitamin D, and steroid hormones to maintain normal metabolism in our body. Cholesterol levels vary by gender and race. White people appear to have a higher cholesterol risk than black people, and women tend to have higher cholesterol levels than men [39]. Kitaharai et al. conducted a prospective study on 1,189,719 Korean adults for 14 years, and they found that high TC was positively correlated with breast cancer in women and prostate cancer and colon cancer in men. Interestingly, it was reported that liver cancer, stomach cancer, and male lung cancer were negatively correlated with TC [40]. In addition, a study suggested that lower TC levels were an independent risk factor for gastric cancer, particularly intestinal-type gastric cancer [41]. It had been reported that higher TC levels were evidently related with lower cancer risk [42]. The NSCLC patients with lower TC had a 61% higher risk of death compared with those with normal TC, which suggested that the serum level of TC may be an independent prognostic indicator for NSCLC overall survival [43]. These studies are in accord with our result that TC levels were lower in the EM group than the NEM group.

Studies have confirmed that pro-inflammatory cytokines and chemokines play a role in lipid metabolism of metabolic diseases [44,45]. It was reported that pro-inflammatory cytokines, chemokines and lipid mediators were the direct causes of plaque rupture [46]. Hilving et al. found that lipids can regulate many genes of pro-inflammatory cytokines and chemokines in TC2 cells, and increase eosinophils to cause asthma [47]. Cytokines are important mediators in the development of cancer [48], for example, TNF-α promotes cancer cachexia by affecting the storage of TG [45]. The NF-κB signaling pathway not only directs inflammatory response through cytokines and other factors to stimulate cell receptors but also related deeply with metabolic disease like obesity and atherosclerosis [49]. Interestingly, NF-κB signaling pathway can also regulate NPC metastasis [50] even can promote recurrence [51]. As mentioned above, those may provide potential mechanisms for the differences of serum lipid levels in male NPC patients with or without eye metastasis, for example, through exosomes or miRNA [51]. The inner mechanisms are thought-provoking, and our team have been studying the risk factors of eye metastasis of many different cancers, we once found high-density lipoprotein can be a sensitive index for eye metastases in colorectal cancer patients [52]. Our study aims to find sensitive serological markers as an auxiliary reference standard for early diagnosis of eye metastasis of nasopharyngeal carcinoma.

Our study was characterized by some limitations. First, the number of male NPC patients in the present study was limited and all of them were from Jiangxi Province, which means the present study may not be applicable to male NPC patients throughout China. Second, EM of NPC is not common, so we only enrolled a few cases. In addition, this was a retrospective study, so memory bias may have been a factor. Moreover, the onset time of the NPC patients was not exactly the same, which might affect the accuracy of the results. Finally, some comparison should be made in weight, BMI index, blood glucose level, whether diabetic or not etc. because abnormal levels of TG and TC are often signs of metabolic disorders. Therefore, further investigations and better research design are needed to confirm our conclusion.

## Conclusion

In summary, our study found that TG and TC can be used as risk factors for EM in male NPC patients, and their cutoff values were 0.975 and 3.425 mmol/l, respectively, with an AUC of 0.764 and 0.681, respectively. These data clarified the diagnostic value of TG and TC in predicting EM in male NPC patients, and they provide evidence for the differential diagnosis between EM and post-radiation eye damage in patients with NPC. Although their sensitivity and specificity were limited, testing the levels of serum lipids is superior in repeatability, low cost, simple, and has a fast operation. It enables patients to monitor the disease and track the risk of EM. By combining it with available diagnostic imaging techniques, it is a promising clinical challenge to develop a more reliable method for diagnosing EM in male NPC patients. Early diagnosis and specific treatment of EM will greatly improve the quality of life of male NPC patients.

## Data Availability

The datasets generated during and/or analyzed during the current study are available from the corresponding author on reasonable request.

## Abbreviations

Apo A1: apolipoprotein A1
Apo B: apolipoprotein B
AUC: area under the curve
EM: eye metastasis
HDL: high-density lipoprotein
LDL: low-density lipoprotein
NEM: non-eye metastasis
NPC: nasopharyngeal carcinoma
ROC: receiver operating curve
TC: total cholesterol
TG: triglyceride
